# Effects of nicosulfuron on plant growth and sugar metabolism in sweet maize (*Zea mays* L.)

**DOI:** 10.1371/journal.pone.0276606

**Published:** 2022-10-21

**Authors:** Ningwei Xu, Zhenxing Wu, Xiangling Li, Min Yang, Jinling Han, Bin Lu, Bingshe Lu, Jian Wang

**Affiliations:** 1 College of Landscape and Tourism, Hebei Agricultural University, Baoding, China; 2 College of Agronomy and Biotechnology, Hebei Key Laboratory of Crop Stress Biology, Hebei Normal University of Science &Technology, Qinhuangdao, China; 3 Institute of Maize and Featured Upland Crops, Zhejiang Academy of Agricultural Sciences, Dongyang, China; United Arab Emirates University, UNITED ARAB EMIRATES

## Abstract

The sulfonylurea herbicide nicosulfuron is efficient, harmless and selective at low doses and has been widely used in maize cultivation. In this study, a pair of corn sister lines, HK301 (nicosulfuron-tolerence, NT) and HK320 (nicosulfuron-sensitive, NS), was chosen to study the effect of nicosulfuron on plant growth and sugar metabolism in sweet maize (*Zea mays* L.) seedlings. All the experimental samples were subjected to treatment with water or 80 mg kg^–1^ of nicosulfuron when the sweet maize seedlings grew to the four-leaf stage. Nicosulfuron significantly inhibited the growth of NS line. The content of sucrose and the activities of sucrose phosphate synthase and sucrose synthase in the two inbred lines increased differentially under nicosulfuron stress compared with the respective control treatment. After nicosulfuron treatment, the activities of hexokinase and 6-phosphofructokinase and the contents of pyruvic acid and citric acid in NS line decreased significantly compared with those of NT line, while the content of sucrose and activities of sucrose phosphate synthase and sucrose synthase increased significantly. The disruption of sugar metabolism in NS line led to a lower supply of energy for growth. This study showed that the glycolysis pathway and the tricarboxylic acid cycle were enhanced in nicosulfuron-tolerant line under nicosulfuron stress in enhancing the adaptability of sweet maize.

## Introduction

The sulfonylurea herbicide nicosulfuron, which is efficient, harmless and selective at low doses, is widely used in agricultural production, particularly in the cultivation of maize [1–3]. The residues of nicosulfuron in the soils and rivers are degraded by hydrolysis and microbial activity, which results in less toxicity to the next crop [4]. Nicosulfuron has been one of the most important herbicides that is widely used in China. It fills a void in the availability of postemergence herbicides in corn.

Acetolactate synthase (ALS) is the target site that responds to nicosulfuron, and sensitive plants cannot grow well when this enzyme is inhibited [5]. Nicosulfuron reduces the synthesis of branched-chain amino acids (BCAAs), including valine, leucine, and isoleucine, while simultaneously inhibiting the activity of ALS and growth of plants cell, thus, killing the plants [6,7]. Nicosulfuron causes phytotoxicity in some plants, which leads to shortened roots, local chlorosis, and yellowing, purple and curly leaves [8]. Since Morton and Harvey [9] discovered that sweet maize was seriously damaged after nicosulfuron was sprayed. Many studies have shown that maize varieties vary significantly in their tolerance to this herbicide [10–12]. Some maize cultivars are sensitive to nicosulfuron. After 7–14 days of treatment, the phytotoxicity caused by nicosulfuron can severely damage sensitive maize seedlings [13–16]. In contrast, tolerant maize cultivars can usually degrade nicosulfuron to inactive compounds or conjugate the herbicide with glucose.

Nicosulfuron is a photosynthetic system inhibitor and can reduce the content of chlorophyll and the ability of the plant to conduct photosynthesis by interfering with the rate of electron transport of the plants [17–19]. Spraying nicosulfuron affects the photosynthetic mechanism by altering the metabolism of plant cells, which results in the disintegration of chloroplasts and a change in the leaf color of plants. The photosynthetic pigments and photosynthetic-related protein activities of plants are significantly reduced, and nicosulfuron destroys the chloroplast structure [20–22]. Thus, the photosynthetic ability to assimilate carbon, absorb light and transmit it, and distribute this energy between photosystem II (PSII) and PSI decrease, which results in the inability to synthesize ATP and NADPH [23]. Our preliminary studies showed that nicosulfuron stress substantially affected the growth of waxy maize (*Zea mays* L.). A combination of nicosulfuron toxicity and nicosulfuron-induced oxidative stress was responsible for the eventual death of susceptible inbred lines [15]. It was confirmed that the mechanism of resistance to nicosulfuron was associated with the rate of photosynthesis, metabolism of reactive oxygen species (ROS), and protective mechanisms [24]. The activity of C_4_ photosynthetic enzymes and expression of key genes are extremely significant in enabling sweet maize plants to withstand nicosulfuron stress at a photosynthetic physiological level with different degrees of resistance to this herbicide [19]. After treatment with nicosulfuron, the nicosulfuron-tolerant maize had significantly higher net photosynthetic rates, transpiration rates, stomatal conductance, leaf maximum photochemical efficiency of PSII, photochemical quenching of chlorophyll fluorescence, and electron transport rates compared with those for nicosulfuron-sensitive maize [22]. The results from our preliminary studies indicate that plants can improve photosynthesis to alleviate the phytotoxic effects of nicosulfuron.

The carbohydrates produced by photosynthesis in plants are either transported to other organs as soluble sugars (glucose, sucrose and fructose) or accumulate in the leaves as soluble sugars and starch and play vital roles in structure and metabolism in all living cells [25,26]. Sucrose is the major product of photosynthesis and since it is related to plant growth, development, storage, signaling, stress acclimation and carbon transfer, it is regarded as the key sugar in plant lifecycles [27,28]. Fructose is an ubiquitous source of carbon and energy for plant cells [29]. As an important osmotic regulatory substance, the accumulation of sugar can help the plants to recover from most abiotic stresses, contribute to osmoregulation and provide protection for biomolecules, which has been correlated with mechanisms of resistance in many plant species [25,30].

Maize is one of the major food crops in China, and it plays an important role in safeguarding food security. Widening the understanding of enzyme activities involved in sugar metabolism will advance our knowledge of maize mechanisms to physiological adaptation and detoxification against various abiotic stresses. Changes in sugar metabolism under abiotic stresses at both the physiological and biochemical levels have been widely reported [31–33]. However, the effects of nicosulfuron as an abiotic stress on maize growth and sugar metabolism have not been reported. In this study, based on our previous research, we investigated the effects of nicosulfuron on plant growth and sugar metabolism in the nicosulfuron-resistant (NT) and -sensitive sister (NS) lines of sweet maize, HK301 and HK320, respectively. The contents of carbohydrates and the activities of key enzymes in glycolysis metabolism and organic acids of the tricarboxylic acid (TCA) cycle were measured. These findings should provide insights into the adaptation and physiological mechanism of drug resistance of sweet maize under herbicide stress.

## Materials and methods

### Experimental materials

A pair of corn sister lines (nicosulfuron-tolerant [NT] HK301 and nicosulfuron-sensitive [NS] HK320), which were developed by Hebei Normal University of Science & Technology (Qinhuangdao City, China), was chosen for this experiment. Paired sister lines were chosen to minimize effects that could be caused by differences in their genetic backgrounds. Nicosulfuron was purchased from De Beier Chemical Co., Ltd., Henan Province, China.

### Experimental design

The field experiments were designed at the Changli Farm of the Hebei Normal University of Science & Technology (39°25’N, 118°45’E), which is within the warm temperate zone and has a monsoon-affected semi-humid continental climate. The fundamental nutrient content of the soil tested was 32.63 g organic matter kg^–1^, 3.24 g total nitrogen kg^–1^, 121.32 mg alkaline hydrolyzed nitrogen kg^–1^, 20.31 mg available phosphorous kg^–1^, and 109.28 mg available potassium kg^–1^.

A two-year herbicide concentration screening test was established in 2018–2019 to ascertain a suitable experimental concentration of nicosulfuron in the field. Three replicates of a split plot experimental design were used with the nicosulfuron treatment defining the main plots and inbred lines within subplots. Each inbred line was sown in 15 rows that were 5 m long with 14.9 cm between plants and 60 cm between rows. The two inbred lines was sown during the first 10 days of May. The average temperature was 14.8 ~ 15.3°C and the sowing depth was 4 cm. Two seeds were placed in each hole to ensure that 500 seedlings per plot were grown. An electric backpack sprayer with a nozzle was used to spray at effective concentrations of 0 (control), 20, 40, 80, 120, 160, 200, 240, 280, and 320 mg kg^–1^ of nicosulfuron when the NT and NS maize seedlings had reached the four-leaf stage. The dose of nicosulfuron was applied at 36 g × hm^-2^. The NT plants grew well at treatment with 80 mg kg^–1^ of nicosulfuron, and 97.51% of the plants survived. In contrast, the NS plants wilted or completely died, resulting in the total loss of plants as the time of exposure was prolonged [22]. Thus, the concentration of 80 mg kg^–1^ was chosen for the rest of experiment.

The field experiment, which was designed as a randomized complete block design with three replicates, was initiated at Changli Farm in 2020. The inbred lines were sown as described above for the preliminary experiment. After 30 days, maize seedlings at the four-leaf stage were sprayed with nicosulfuron at an effective concentration of 80 mg kg^–1^ with water used as the control. The data and samples were collected at 1, 3, 5, and 7 days after treatment (DAT) with nicosulfuron. Seedling leaves were sampled for determination of physiological and biochemical data.

### Determination of the plant height, main root length, and adventitious root number

Nicosulfuron was used to treat the maize seedlings when the fourth leaves were fully developed. A total of 30 replicates of the plant height, main root length and adventitious root number were determined at 1, 3, 5, and 7 DAT. The plant height and main root length were determined by a ruler that was accurate to 0.1 cm. The adventitious roots which were greater than 8.0 cm in length were manually counted.

### Determination of the contents of sucrose and fructose

The contents of sucrose and fructose were determined using the assay kits ZHT-2-Y and GT-2-Y, respectively (Suzhou Comin Biotechnology Co., Ltd., Suzhou, China) according to the manufacturer’s instructions. The contents were assayed at 480 nm on a UV- 2600 spectrophotometer (Shimadzu, Kyoto, Japan). The contents of sucrose and fructose were calculated and expressed in mg g^-1^ of fresh weight (FW).

### Determination of the activities of enzymes involved in the synthesis of sucrose

The activities of sucrose phosphate synthase (SPS) and sucrose synthase (SS) were assayed using a kit for each enzyme (SPS-2-Y, and SSⅡ-2-Y, respectively) (Suzhou Comin Biotechnology Co., Ltd.) according to the manufacturer’s instructions. A total of 0.1 g of leaf samples were ground into homogenate in a mortar. The homogenate was transferred into a centrifuge tube and centrifuged at 8000 g, 4°C for 10 min. These enzymes were assayed at 480 nm on a UV-2600 spectrophotometer as described above and expressed in μg min^-1^ g^-1^ (FW).

### Determination of the activities of enzymes involved in the glycolytic pathway

The activities of hexokinase (HK) and 6-phosphofructokinase (PFK) were determined using assay kits for each respective enzyme (HK-2-Y, and PFK-2-Y, respectively) (Suzhou Comin Biotechnology Co., Ltd.) according to the manufacturer’s instructions. A total of 0.1 g leaf samples were ground into homogenate in a mortar. The homogenate was transferred into a centrifuge tube and centrifuged at 8000 g, 4°C for 10 min. The enzymes were assayed at 340 nm on a UV-2600 spectrophotometer (Shimadzu) at 340 nm. The enzyme activities were calculated and expressed in nmol min^-1^ g^-1^ (FW).

### Determination of the major organic acids involved in the TCA Cycle

The contents of pyruvic acid (PA) and citric acid (CA) were measured using the assay kits PA-2-Y and CA-2-W, respectively, according to the manufacturer’s instructions (Suzhou Comin Biotechnology Co., Ltd.). A total of 0.1 g leaf samples were ground into a homogenate in a mortar. The homogenate was transferred into a centrifuge tube and centrifuged at 8000 g, 25°C, 10 min for PA and 11000 g, 4°C, 10 min for CA, and assayed at 520 nm and 545 nm for PA and CA on a UV-2600 spectrophotometer, respectively (Shimadzu). The PA and CA contents were calculated and expressed in μg g^-1^ (FW) and nmol g^-1^ (FW), respectively.

### Statistical analysis

The data were processed and mapped using Microsoft Excel 2013 (Redmond, WA, USA). A one-way analysis of variance (ANOVA) and the mean values were compared using the least significant difference (LSD) test in SPSS 21.0 (IBM, Inc., Armonk, NY, USA). Significant differences were identified at the P < 0.05 threshold.

## Results

### Plant height, main root length, and adventitious root number

As shown in Table 1, an increase in the time of exposure was accompanied by a significant increase in the plant height, main root length and adventitious root number in the NT line at both the CK and 80 mg kg^–1^ nicosulfuron levels. The plant height in NT-CK at 1, 3, 5, and 7 DAT increased by approximately 5.4%, 12.2%, 18.0% and 22.3%, respectively, compared with that at 0 DAT. In contrast, the plant height in NT increased by only 1.8%, 6.7%, 10.6% and 14.5% at 1, 3, 5, and 7 DAT, respectively, compared with that at 0 DAT. The main root lengths in the NT-CK treatments at 1, 3, 5, and 7 DAT increased by approximately 9.7%, 16.4%, 21.8% and 33.6%, respectively, compared with those at 0 DAT. In contrast, the main root length in NT increased by only 3.0%, 9.5%, 16.8% and 22.8% at 1, 3, 5, and 7 DAT, respectively, compared with that at 0 DAT. The adventitious root number in NT-CK at 1, 3, 5, and 7 DAT increased by approximately 9.1%, 27.3%, 45.5% and 54.5%, respectively, compared with that at 0 DAT. Alternatively, the adventitious root number in NT increased by only 0.0%, 8.3%, 16.7% and 25.0% at 1, 3, 5, and 7 DAT, respectively, compared with that at 0 DAT. Two distinctly different trends of phenotypic data were found in NS-CK and NS. The plant height, main root length, and adventitious root number in NS-CK increased continuously at 0, 1, 3, 5 and 7 DAT, while these parameters decreased significantly at 3, 5, and 7 DAT in NS.

**Table 1:** Effect of nicosulfuron on plant height, main root length, and adventitious root number of sweet maize seedlings.

Exposure time [d]	Plant height [cm]				Main root length [cm]				Adventitious root number			
	NT-CK	NT	NS-CK	NS	NT-CK	NT	NS-CK	NS	NT-CK	NT	NS-CK	NS
0	9.27 ± 0.33 e	9.43 ± 0.25 d	8.57 ± 0.34 e	8.47 ± 0.26 a	7.93 ± 0.26 d	7.73 ± 0.26 c	7.37 ± 0.41 d	7.23 ± 0.54 a	3.67 ± 0.47 c	4.00 ± 0.00 c	3.67 ± 0.47 c	3.67 ± 0.47 a
1	9.77 ± 0.34 d	9.60 ± 0.29 d	9.13 ± 0.31 d	8.50 ± 0.22 a	8.70 ± 0.51 c	7.97 ± 0.25 bc	7.80 ± 0.36 cd	7.30 ± 0.51 a	4.00 ± 0.00 c	4.00 ± 0.00 c	4.00 ± 0.00 bc	3.67 ± 0.47 a
3	10.40 ± 0.51 c	10.07 ± 0.25 c	9.73 ± 0.05 c	7.93 ± 0.29 b	9.23 ± 0.31 bc	8.47 ± 0.45 b	8.27 ± 0.39 c	6.67 ± 0.42 b	4.67 ± 0.47 b	4.33 ± 0.47 bc	4.33 ± 0.47 abc	3.33 ± 0.47 a
5	10.93 ± 0.40 b	10.43 ± 0.21 b	10.30 ± 0.08 b	7.03 ± 0.26 c	9.67 ± 0.31 b	9.03 ± 0.17 a	9.03 ± 0.25 b	6.23 ± 0.39 c	5.33 ± 0.47 a	4.67 ± 0.47 ab	4.67 ± 0.47 ab	3.00 ± 0.00 a
7	11.33 ± 0.33 a	10.80 ± 0.08 a	10.70 ± 0.08 a	6.27 ± 0.45 d	10.60 ± 0.22 a	9.50 ± 0.16 a	9.73 ± 0.05 a	5.87 ± 0.40 d	5.67 ± 0.47 a	5.00 ± 0.00 a	5.00 ± 0.00 a	3.00 ± 0.00 a

NT-CK: Water treatment in HK301; NT: Nicosulfuron 80 mg kg^–1^ treatment in HK301; NS-CK: Water treatment in HK320; NS: Nicosulfuron 80 mg kg^–1^ treatment in HK320. The values are means ± standard errors (n = 30) of experimental data. Different lowercase letters in the same column indicate significant differences at the *P* < 0.05 level.

### Contents of sucrose and fructose

**Fig 1:**
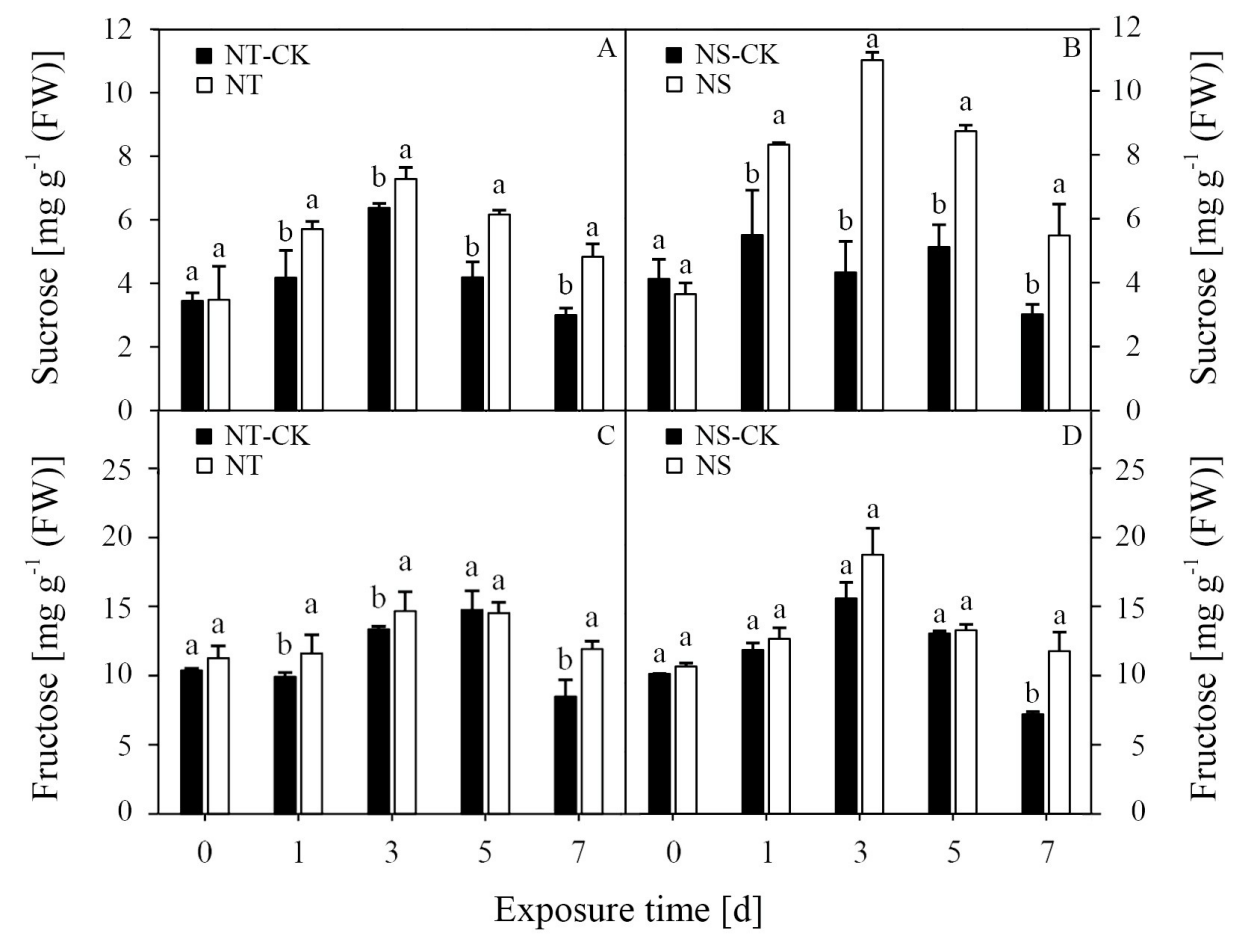
Effects of nicosulfuron on the sucrose (A, B) and fructose (C, D) in leaves of sweet maize seedlings. NT-CK: Water treatment in HK301; NT: Nicosulfuron 80 mg kg^–1^ treatment in HK301; NS-CK: Water treatment in HK320; NS: Nicosulfuron 80 mg kg^–1^ treatment in HK320. Vertical bars represent the SE (n = 3). Small letters (a, b) indicate differences between values obtained on different days after nicosulfuron treatment (*P* < 0.05) according to a least significant difference (LSD) test.

Treatment with nicosulfuron resulted in significant changes in the content of sucrose of both inbred lines. Compared with NT-CK, the content of sucrose in NT increased by approximately 36.6%, 14.2%, 47.1%, and 61.5% compared with that at 1, 3, 5, and 7 DAT, respectively. Treatment with nicosulfuron significantly increased the content of sucrose in NS at 1, 3, 5, and 7 DAT by 51.5%, 153.7%, 71.0%, and 81.7%, respectively, compared with the NS-CK. Compared with each CK, the increment of sucrose in the NS line was higher than that of the NT line at 1, 3, 5, and 7 DAT (Fig 1A and 1B).

Nicosulfuron treatment significantly increased the content of fructose in the NT line at 1, 3, and 7 DAT by 17.0%, 10.0%, and 40.5%, respectively, compared with the NT-CK (Fig 1C). In addition, after nicosulfuron treatment, the content of fructose in the NS line significantly increased at 7 DAT compared with the NS-CK. Compared with each CK, the increment of fructose in the NS line was higher than that of the NT line at 3, 5, and 7 DAT (Fig 1D).

### Activities of SPS and SS

**Fig 2:**
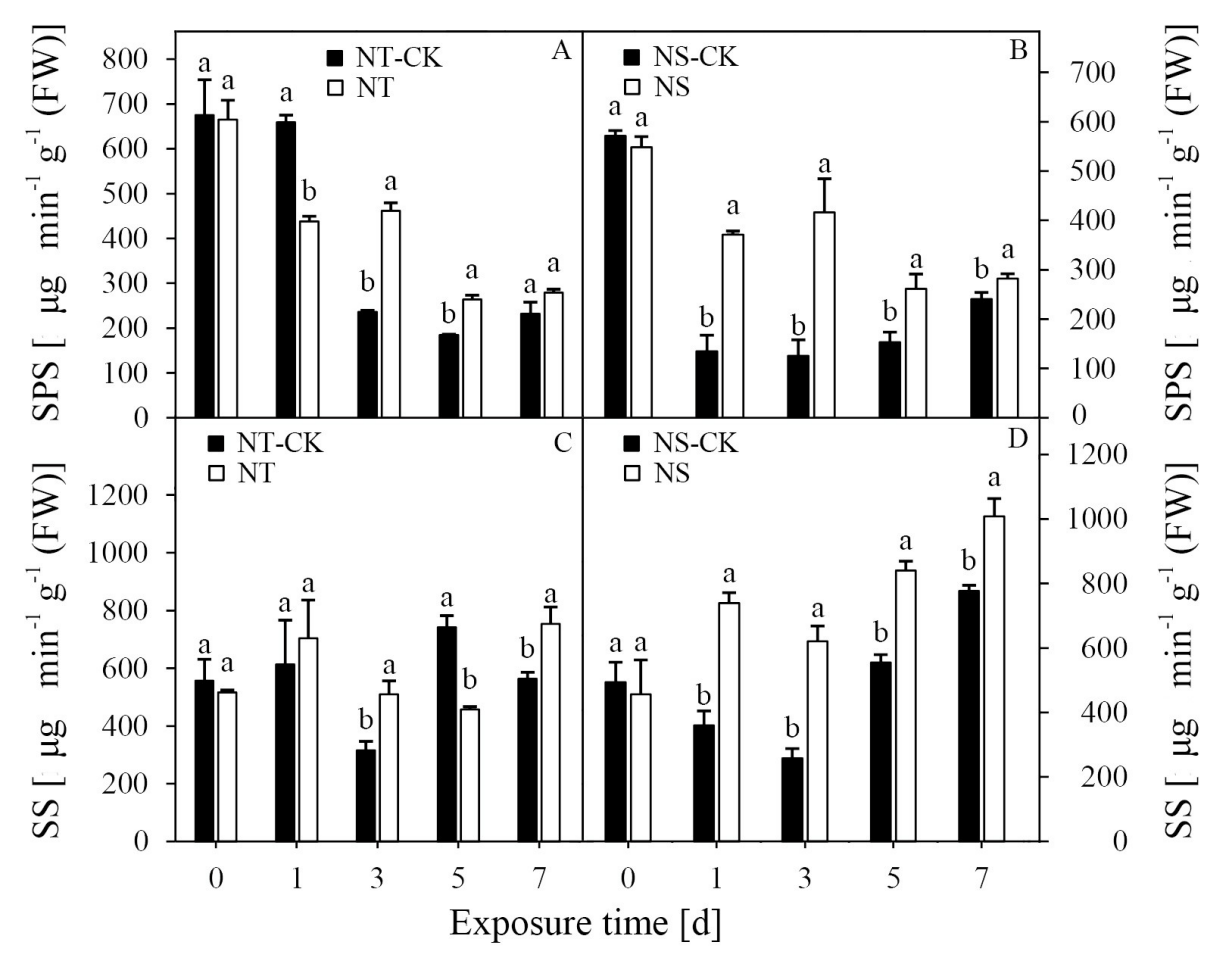
Effects of nicosulfuron on the sucrose phosphate synthase (SPS) (A, B), and sucrose synthase (SS) (C, D) in leaves of sweet maize seedlings. NT-CK: Water treatment in HK301; NT: Nicosulfuron 80 mg kg^–1^ treatment in HK301; NS-CK: Water treatment in HK320; NS: Nicosulfuron 80 mg kg^–1^ treatment in HK320. Vertical bars represent the SE (n = 3). Small letters (a, b) indicate differences between values obtained on different days after nicosulfuron treatment (*P* < 0.05) according to a least significant difference (LSD) test (the same below).

Aside from the results at 7 DAT, treatment with nicosulfuron significantly affected the activity of SPS in the NC line. Compared with NT-CK, this treatment significantly increased the activity of SPS in the NT line at 3 and 5 DAT by 96.0% and 43.0%, respectively (Fig 2A). However, the activity of SPS in the NS line was significantly higher than that of the control at all time points. The SPS activity in the NS line increased significantly at 1, 3, 5, and 7 DAT by 175.0%, 232.2%, 70.3%, and 17.3%, respectively, compared with the NS-CK (Fig 2B).

Except at 5 DAT, the activity of SS in the NT line increased at 1, 3, and 7 DAT, which was 314.7%, 61.4%, and 33.8% higher than those of the control, respectively (Fig 2C). In comparison, under herbicide treatment, the activity of SS in the NS line increased significantly at 1, 3, 5, and 7 DAT by 105.5%, 140.6%, 51.4%, and 29.9%, respectively, compared with the NS-CK (Fig 2D).

### Activities of HK and PFK

**Fig 3:**
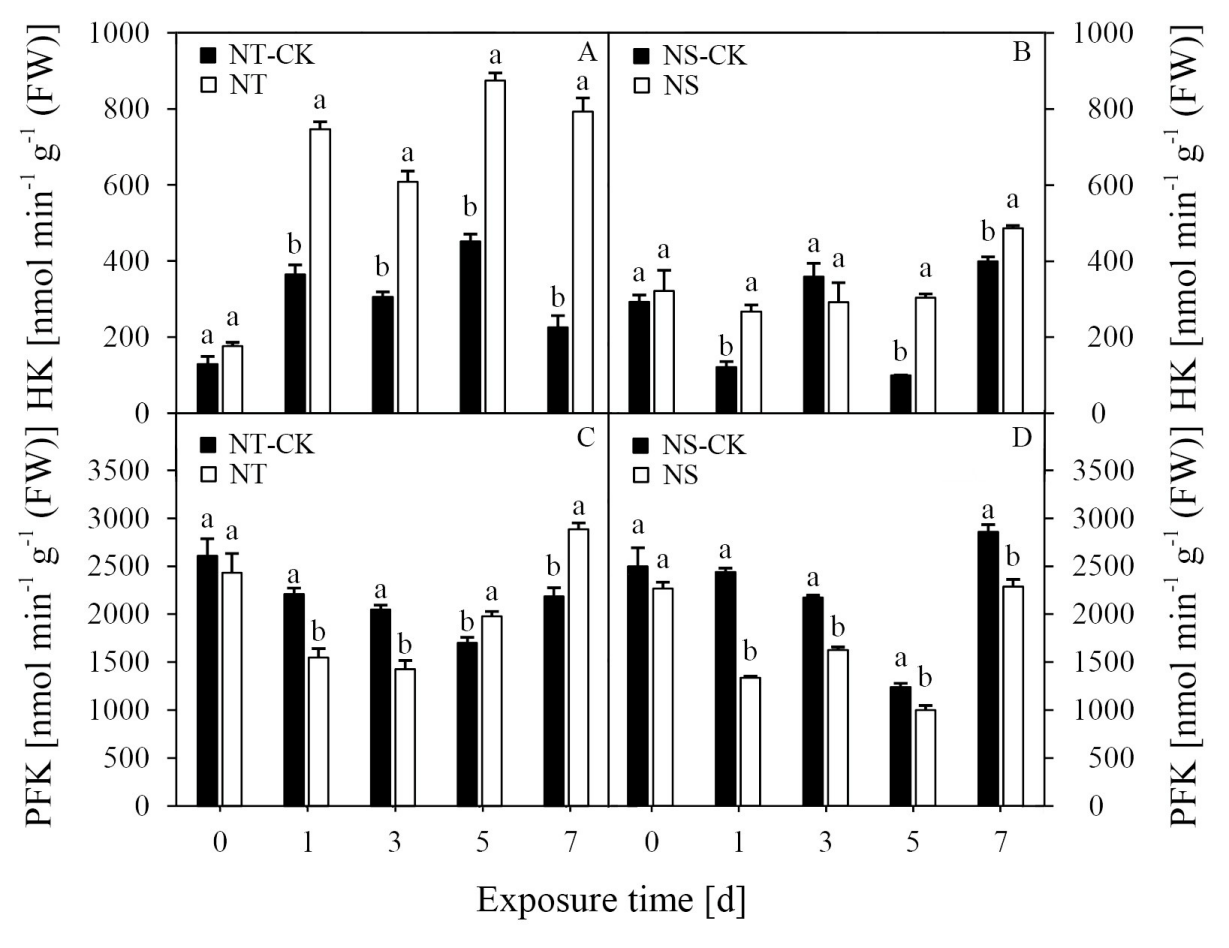
Effects of nicosulfuron on the hexokinase (HK) (A, B), and phosphofructokinase (PFK) (C, D) in leaves of sweet maize seedlings.

The HK activity of the two inbred lines responded differently as the herbicide exposure time progressed (Fig 3A and 3B). After 1 DAT, the HK activity of the NT line increased significantly and was maintained at a higher level. The activity of HK in the NT was significantly higher than that of the control under nicosulfuron stress at all time points. Compared with the NT-CK, the treatment with nicosulfuron significantly increased the HK activity of the NT line at 1, 3, 5, and 7 DAT by up to 104.4%, 99.1%, 93.5%, and 251.7%, respectively. However, treatment with nicosulfuron significantly increased the HK activity of the NS line except at 3 DAT. The HK activity in NS increased at 1, 5, and 7 DAT by 120.5%, 205.1%, and 21.7%, respectively, compared with the NS-CK. In addition, the average HK activity of the NT line was higher than that of the NS line.

After nicosulfuron was sprayed, the PFK activity of the NT line was lower than that of the CK at 1 and 3 DAT by 29.9% and 30.3%, respectively. Compared with the NT-CK, the treatment with nicosulfuron significantly increased the PFK activity of the NT line at 5 and 7 DAT by 16.3% and 32.1%, respectively (Fig 3C). However, the PFK activity of NS at 1, 3, 5, and 7 DAT was significantly lower under nicosulfuron stress than under each control condition and decreased by 45.2%, 25.2%, 19.2%, and 20.0%, respectively (Fig 3D).

### Contents of PA and CA

**Fig 4:**
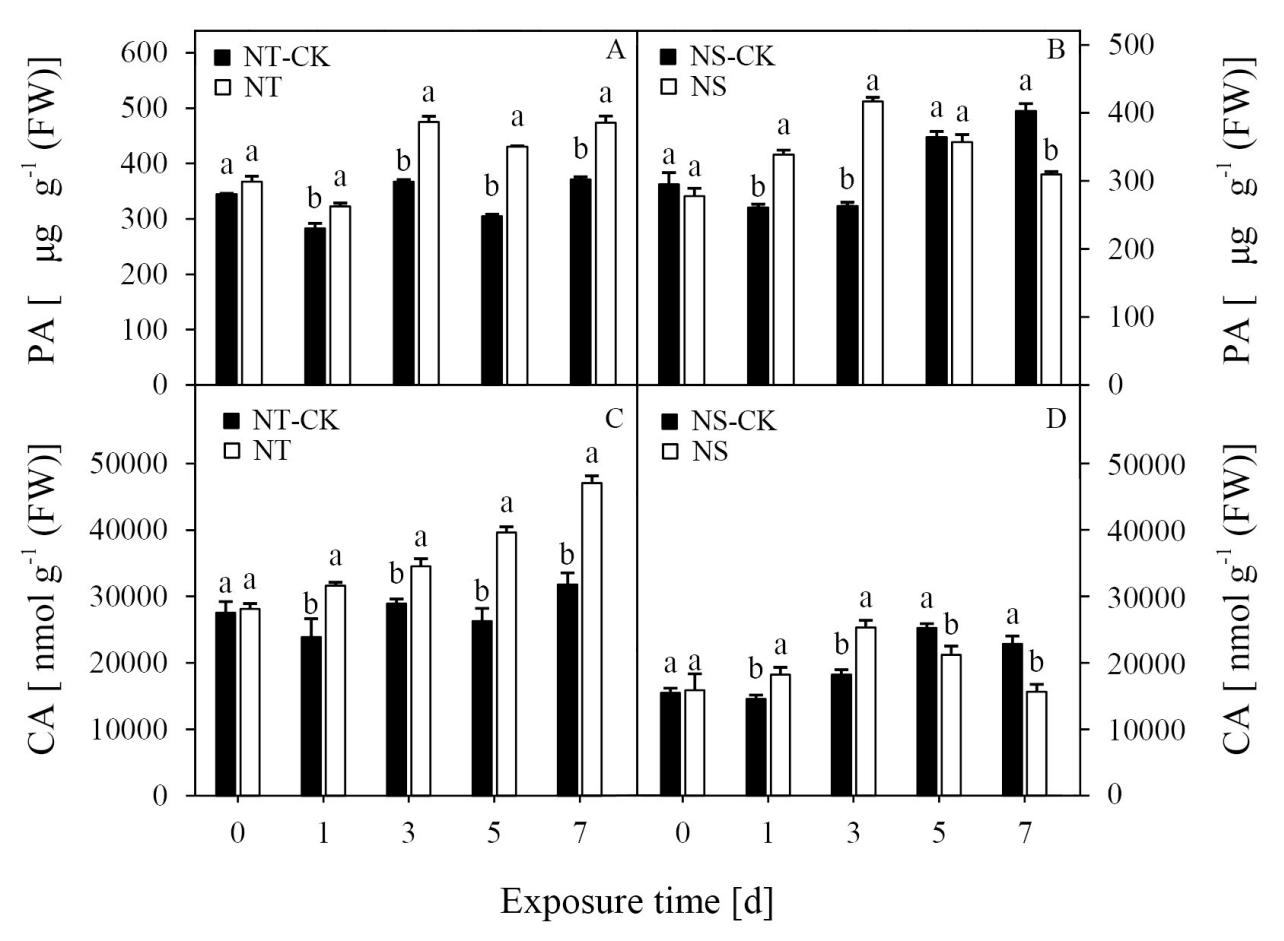
Effects of nicosulfuron on the pyruvic acid (PA) (A, B), and citric acid (CA) (C, D) in leaves of sweet maize seedlings.

The contents of PA and CA were identified in the two inbred lines responded differently after nicosulfuron was sprayed. Under the nicosulfuron treatment, the content of PA in the NT line was the highest at 3 DAT and remained at a high level without a significant change, while the content of PA of the NS increased significantly at 1 and 3 DAT. Compared with the NT-CK, treatment with nicosulfuron significantly increased the content of PA of the NT line at 1, 3, 5, and 7 DAT by 14.1%, 29.4%, 41.2%, and 27.6%, respectively (Fig 4A). In contrast, the content of PA of the NS line reached its maximum value after 3 DAT and subsequently decreased. Compared with NS-CK, the treatment with nicosulfuron significantly increased the content of PA in the NS line at 1 and 3 DAT by 29.7%, and 58.5%, respectively. The content of PA in the NS line was 23.2% lower than that of the control at 7 DAT (Fig 4B).

Treatment with nicosulfuron induced a significant increase in the content of CA in the NT line (Fig 4C). However, the content of CA in the NS line increased first and then decreased with the time of exposure (Fig 4D). Under herbicide treatment, the content of CA in the NT line significantly increased at 1, 3, 5, and 7 DAT by 32.5%, 19.3%, 50.7%, and 48.1%, respectively, compared with the NT-CK. Compared with the NS-CK, treatment with nicosulfuron significantly increased the content of CA of the NS line at 1 and 3 DAT by 25.1%, and 38.8%, respectively. In contrast, the CA content of the NS line was significantly reduced at 5 and 7 DAT, which was 16.1% and 31.6% lower than the control, respectively.

## Discussion

### Inhibition of plant growth caused by nicosulfuron

Many studies have shown that nicosulfuron can inhibit seed germination and crop growth [9,15,34]. The reduction in growth under nicosulfuron stress could be partly owing to the reduction in photosynthetic activity as indicated by our earlier findings [24], which results in lower stomatal conductance and limits the assimilation of CO_2_. In addition, numerous studies have shown that plants can accumulate an array of metabolites under environmental stresses, particularly amino acids. A large accumulation of the BCAAs was observed in plants subjected to osmotic stress, suggesting that the BCAAs could play an important role in stress responses [35,36]. In this study, we found that the plant height, root length and root number of the two inbred lines were inhibited to varying degrees with nicosulfuron stress, suggesting that the NT and NS lines differentially tolerate nicosulfuron. The reduction in growth under nicosulfuron stress is likely owing to the reduction in photosynthetic activity and oxidative stress as indicated by our earlier findings [15,24]. Furthermore, nicosulfuron also inhibited the synthesis of BCAAs in sensitive plants, which led to the changes in osmotic stress and sugar metabolites in the NS line.

### Modulation of sugar accumulation by nicosulfuron

The rate of degradation of nicosulfuron in the resistant plants was higher than that in the susceptible plants, while the residual nicosulfuron in susceptible plants will persist as a toxic factor and induce osmotic stress and ROS [10,37]. The subsequent increase in antioxidative capacity protected the nicosulfuron-resistant maize from the severe damage caused by nicosulfuron stress [22]. Osmotic stress and the accumulation of ROS could also be associated with the accumulation of soluble sugar, which has generally been considered to be an adaptive response to the nicosulfuron stress [38]. Our results confirmed that nicosulfuron significantly increased the content of sucrose of the two inbred lines compared with their respective control. This could be because sugar accumulated as a result of the formation of ROS by nicosulfuron exposure. The accumulation of sugars has been related to the mechanisms that result in resistance to stress in many plants. Das et al. [25], who studied rice (*Oryza sativa*), described how salt stress in the test seedlings resulted in an increase in the contents of both reducing and non-reducing sugars for basal metabolism [25]. Other current studies demonstrated that the sucrose content increased in the leaves of Madagascar periwinkle (*Catharanthus roseus*), sugar beet (*Beta vulgaris* L. cv F58-554H1) and maize under abiotic stresses [39–41]. The accumulation of sugars in the NS line was higher than that in the NT line, which could contribute to the sensitivity to nicosulfuron that limits the growth of maize seedlings and could likely be determined by the decrease in sucrose transport capacity under nicosulfuron stress [42]. Although a large accumulation of carbohydrates enhances metabolism, it is also a vital factor that inhibits photosynthesis [43].

SPS is of primary importance to the catalysis of sucrose phosphate, which plays an essential role in the biochemical regulation of sucrose formation [44,45]. SS is a bidirectional reactive enzyme that catalyzes both the synthesis and decomposition of sucrose and is one of the key enzymes in sucrose metabolism [29,46]. The activity of SPS was observed and induced in spinach (*Spinacea oleracea*), peach (*Prunus persica*) and tomato (*Solanum lycopersicon*) under abiotic stresses, including water stress and salinity [47–49]. In this study, nicosulfuron significantly increased the activities of SPS and SS of the two inbred lines overall compared with their respective control, supporting the increase in sucrose as reported earlier.

### Modulation of sugar metabolism by nicosulfuron

Nicosulfuron inhibits the activity of ALS in sensitive plants [50], which leads to the etiolation and wilting of maize leaves. Thus, this constricts the supply of carbohydrates, which results in a decrease in physiological and metabolic activities as a result of the growth restriction in maize under nicosulfuron stress. HK and PFK are critical to the glycolysis pathway and have irreversible activity. HK catalyzes the conversion of glucose to glucose-6-phosphate, which results in the formation of fructose-6-phosphate under the action of phosphoglucose isomerase and the phosphorylation of fructose-1, 6-diphosphate under the action of PFK. Singla et al. [32] observed a distinctly different response in the activities of HK and PFK in the roots and leaves of different cultivars of sorghum (*Sorghum bicolor*). The HK activity in the two cultivars of *S*. *bicolor* all increased in their roots. The PFK activity of the tolerant cultivar increased during flooding, while the leaves of sensitive cultivar were found to have lower PFK activity than that of their respective controls. Our research has shown that the HK activity increased observably in the leaves of the NT line compared with those of the NT-CK line, which was considerably greater than those in the NS line. The PFK activity of the NT line increased significantly at 5 and 7 DAT, while it decreased noticeably after nicosulfuron treatment in NS compared with their respective controls. The activities of enzymes that are involved in the glycolysis metabolic pathway appeared to have increased in the NT line, particularly at 5 and 7 DAT, which suggests that the NT line was more strongly protected against the nicosulfuron stress. Thus, the glucose could be degraded to reduce the osmotic potential, thus, improving the water absorption and metabolism capacity of cells. The metabolism of sugar in sweet maize owing to nicosulfuron is partially related to the involvement of these enzymes.

PA and CA are the final and intermediate products of the glycolysis pathway and the TCA cycle, respectively, which could evaluate the relative intensity of glycolysis pathway. PA plays an important role in enhancing salt tolerance in barley (*Hordeum vulgare*) [51]. The increase in CA supplies more substrate for the mitochondrial citric dehydrogenase and alleviates the oxidative damage caused by abiotic stress and regulation of mitochondrial oxidative balance [33]. In this study, compared with the NT-CK line, nicosulfuron significantly increased the contents of PA and CA in the NT line, demonstrating the increase in enzyme activities related to glycolysis in the NT line as reported earlier. In contrast, the contents of PA and CA decreased at 5 and 7 DAT in the NS line, respectively, compared with the NS-CK line, indicating that the TCA cycle was inhibited by exposure to nicosulfuron. Thus, sugar metabolism was enhanced in nicosulfuron-tolerant maize, which meant that more ATP could be released to help this maize resist the environmental stress of nicosulfuron.

## Conclusions

**Fig 5:**
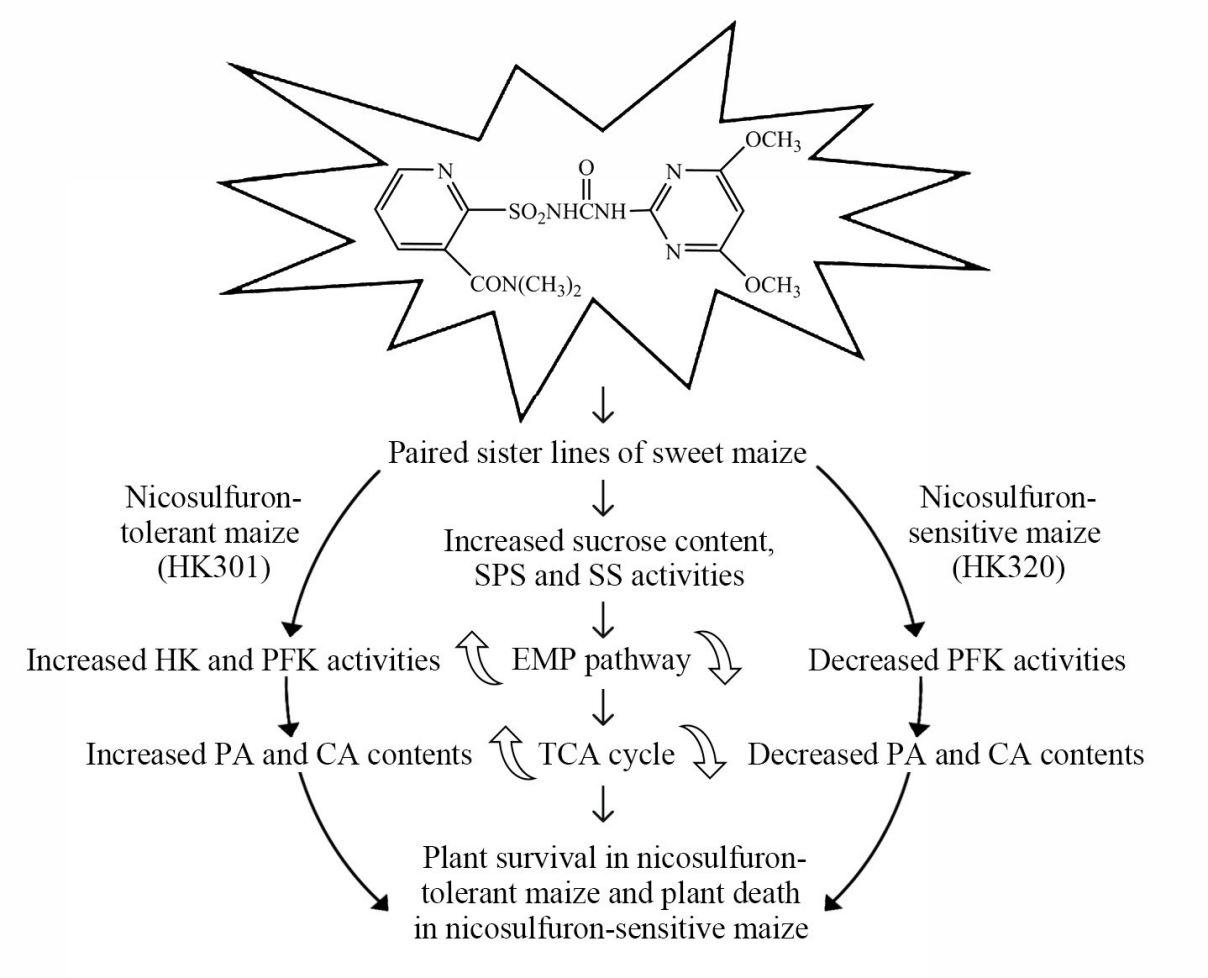
Effects of nicosulfuron on nicosulfuron-tolerant and nicosulfuron-sensitive maize.

The influence of nicosulfuron on plant growth and sugar metabolism of sweet maize from a pair of corn sister lines was comprehensively assessed. The phenotypic data indicated that nicosulfuron inhibited the growth of the two inbred lines at different levels, which had a greater influence on the nicosulfuron-sensitive line. The accumulation of sucrose is important for seedlings to reduce the damage of nicosulfuron. After nicosulfuron treatment, the activities of HK and PFK and the contents of PA and CA of the NS line decreased significantly compared with those of the NT line, while the content of sucrose and the activities of SPS and SS increased significantly (Fig 5). The disruption of sugar metabolism in the NS line led to a lower energy supply for growth. Our study showed that the glycolysis pathway and the TCA cycle were enhanced in the NT line under nicosulfuron stress in enhancing the adaptability of sweet maize.

## Supporting information

S1 File (PDF)

S1 Data (XLSX)

## Abbreviations

ALS: acetolactate synthase
BCAAs: branched-chain amino acids
SPS: sucrose phosphate synthase
SS: sucrose synthase
HK: hexokinase
PFK: 6-phosphofructokinase
PA: pyruvic acid
CA: citric acid
TCA: tricarboxylic acid
ROS: relative oxygen species
